# Evaluation of indirect methods for estimating body fat in dogs using electrical bioimpedance and morphometry

**DOI:** 10.2478/joeb-2026-0009

**Published:** 2026-07-28

**Authors:** Daniel Montoya-Barbosa, Clara Helena González-Correa, William Narváez-Solarte

**Affiliations:** Master in Veterinary Sciences, Universidad de Caldas, Manizales, Colombia; Department of Basic Sciences, Universidad de Caldas, Manizales, Colombia; Department of Animal Health, Research Group in Nutrition, Metabolism and Food Security, Universidad de Caldas,Colombia

**Keywords:** Electrical bioimpedance, canine obesity, body composition, body fat, morphometry, phase angle

## Abstract

Canine obesity causes metabolic issues and reduces quality of life. Assessing adiposity remains challenging due to limited indirect and reference methods. Electrical bioimpedance (BIA) is a proposed tool, but agreement between morphometric and BIA equations, and the body condition score (BCS) role, remains poorly explored in heterogeneous canine populations.

**Objective:**

To evaluate agreement among indirect methods (BCS, morphometry, BIA) for estimating canine body fat and assess BIA's viability for nutritional evaluation.

**Materials and methods:**

A cross-sectional study included 17 healthy dogs from Villamaría, Colombia. Body condition was assessed using 5- and 9-point scales. Morphometric variables and single-frequency 50 kHz BIA parameters (resistance, reactance, phase angle) were recorded. Body fat percentage (%BF) was estimated using four established equations. Method agreement was evaluated via Bland–Altman analysis and Lin's concordance correlation coefficient (CCC). Differences in bioelectrical parameters and lipid profiles between obese and lean dogs were analyzed using independent t-tests.

**Results:**

Morphometric methods showed the highest agreement (CCC = 0.759). Conversely, comparisons involving BIA-derived equations exhibited low or negative agreement, with Bland–Altman analyses revealing wide limits of agreement. Obese dogs had higher resistance and lower phase angles than lean dogs, though differences were not statistically significant (p > 0.05). This lack of significance must be interpreted cautiously due to the small sample size, particularly the obese group (n = 4). HDL cholesterol was significantly lower in obese dogs (p = 0.030), with no differences in total cholesterol.

**Conclusion:**

The evaluated indirect methods showed poor agreement and are not clinically interchangeable. Without a reference method like DEXA, accuracy cannot be determined. While raw BIA parameters aligned physiologically with body condition, their use remains preliminary and requires validation in larger, reference-controlled studies.

## Introduction

Canine obesity is currently the most frequent nutritional disorder in small animal veterinary practice and is associated with metabolic alterations that compromise quality of life and life expectancy. Its proper diagnosis requires the quantification of body adiposity, since body weight alone does not allow differentiating between fat mass (FM) and fat-free mass (FFM), especially in a species characterized by high morphological variability [1,2].

Reference methods for the evaluation of body composition in dogs, such as dual-energy X-ray absorptiometry (DEXA) and deuterium dilution, offer high accuracy; however, their use in veterinary clinical practice is limited due to their cost, technical complexity, and low availability [3,4]. This has driven the development of indirect methods such as the body condition score (BCS), morphometry, and electrical bioimpedance (BIA), which present advantages in terms of application, but also limitations in terms of accuracy, reproducibility, and applicability in dog populations of different breeds [5].

BIA has been proposed as a promising tool for estimating body composition due to its non-invasive nature, low cost, and ease of use [6]. Its foundation is based on the electrical properties of biological tissues, particularly on the relationship between body water content and electrical conductivity. From the measurement of impedance, it is possible to obtain parameters such as resistance (R) and reactance (Xc), from which the phase angle (PhA) is derived, considered an indirect indicator of cellular integrity and the distribution of body fluids [7].

Nevertheless, the application of BIA in dogs faces significant limitations. The predictive equations used to estimate body fat percentage have been developed in specific populations, generally under controlled experimental conditions, which restricts their validity in real clinical contexts [8]. This limitation is particularly relevant in mixed-breed canine populations, such as those predominant in Colombia, where variability in size, body conformation, and adipose tissue distribution can affect the accuracy of the estimates [9,10].

Additionally, the agreement between BIA and other indirect methods such as morphometry and BCS has not been sufficiently explored in Colombian populations, which limits the interpretation of the results obtained using these tools. In this context, the analysis of raw bioelectrical parameters of R, Xc, and PhA, independent of predictive equations, has emerged as an alternative of interest, offering more complete information about the physiological state of the dog [11,12].

The objective of the present study was to evaluate the agreement between the morphometric and electrical bioimpedance-derived equations used for estimating body fat in dogs, as well as to analyze the role of the body condition score as a clinical classification variable and as an input for the BIA-based equations, and to examine the relationship of the raw bioelectrical parameters with body condition. We hypothesized that the results derived from BIA could constitute a complementary alternative for the nutritional assessment of the canine population.

## Materials and methods

### Study design

A cross-sectional observational study was conducted, in which each animal was evaluated at a single time point. This study was considered exploratory and hypothesis-generating; therefore, a convenience sample was used.

### Population and study area

The study was carried out in the municipality of Villamaría, Caldas, Colombia. Seventeen clinically healthy dogs (*Canis lupus familiaris*), over one year of age, of both sexes, belonging to volunteer owners, were included. Inclusion criteria were clinically healthy animals, according to veterinary assessment. Participants were subjected to a minimum 12-hour fast prior to measurements. No restrictions were established by breed, size, reproductive status, or body condition.

### Body condition score assessment

Body condition was evaluated by a single trained observer in order to reduce inter-observer variability. Two scoring systems were used: the 9-point scale (1–9) and the 5-point scale (1–5) [13]. The evaluation was performed through visual inspection and palpation of the ribs, spinous processes, abdominal waist, and tail base. Values were recorded as integers for the 9-point scale. Dogs with a body condition score ≥ 6/9 were classified as overweight or obese. For the 5-point scale, decimal values were used. Individuals with a BCS score ≥ 4/5 were considered over-weight or obese.

### Morphometric measurements

Measurements were performed once per animal, with the dog in a standing position, using a flexible tape measure and a digital scale. The following variables were recorded: body weight (BW); leg length (LL), measured from the middle of the patella to the calcaneal tuberosity; abdominal circumference (AC), measured at the level of lumbar vertebrae L5 and L6; thoracic circumference (TC), measured at the level of the ninth rib corresponding to the xiphoid process; height at the shoulder, measured from the ground to the highest point of the withers; and total body length (TL), measured from the external occipital protuberance to the tail base. All measurements were recorded in kilograms (kg) or centimeters (cm), as appropriate.

### Electrical bioimpedance

**Figure 1.**
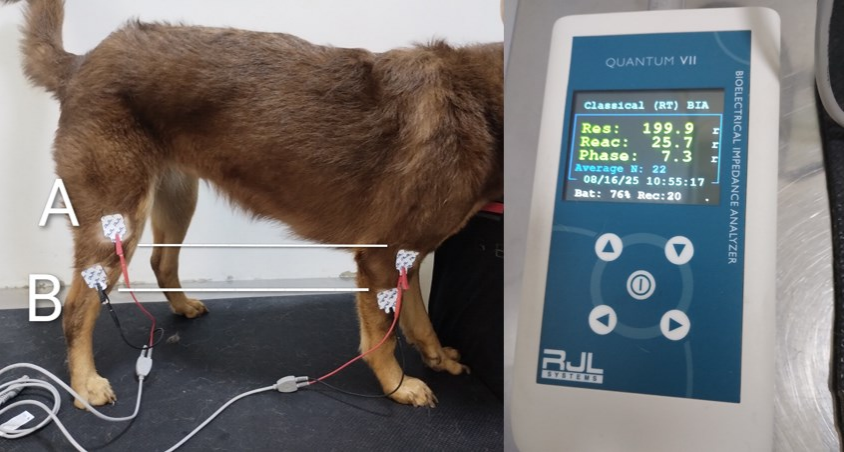
(Left) Placement of voltage electrodes (A), current electrodes (B). (Right) Values of R, Xc, and PhA provided by the BIA device. Source: own elaboration.

Body composition was evaluated using single-frequency electrical bioimpedance (SF-BIA), using a Quantum VII® single-frequency analyzer (RJL Systems, USA), operating at a frequency of 50 kHz. The equipment was previously calibrated according to the manufacturer's instructions, and a tetrapolar configuration was used. During the measurement, the animals were positioned standing on a non-conductive surface, avoiding manual contact. Shaving and skin cleaning with 70% alcohol were performed, and four adhesive electrodes were placed: the voltage electrodes on the cranial region of the right elbow and right stifle, and the current electrodes 7 cm distal to these on the same limb. It should be noted that the electrode placement protocol used has not been formally validated for use in conscious, standing dogs, unlike the studies on which methods 3 and 4 are based, which were conducted under sedation. Furthermore, bioimpedance measurements were taken only once per animal, without repetition or averaging of values, precluding the evaluation of intra-individual repeatability of the technique under the conditions of the present study. Resistance values in ohms (Ω), reactance (Ω), and phase angle in degrees were recorded, as shown in Figure 1.

### Lipid profile

Blood samples were obtained by venipuncture of the cephalic vein, collecting 2 mL of blood in tubes without anticoagulant, with subsequent centrifugation to obtain serum. For the measurement of HDL cholesterol, the HDL Directo Plus 3® reagent (GT Lab, Argentina) was used, based on a direct method, while total cholesterol was determined using the Cholesterol MR® reagent (Linear Chemicals, Spain). The results were expressed in mg/dL.

### Estimation of body fat percentage

Body fat percentage (%BF) was estimated using four methods previously validated in the literature [3,5]. Method 1 corresponded to a sex-dependent morphometric equation based on leg length (LL) and abdominal circumference (AC); for males: %BF = (−1.4 x LL) + (0.77 x AC) + 4, and for females: %BF = (−1.7 x LL) + (0.93 x AC) + 5. Method 2 corresponded to a general morphometric equation based on abdominal circumference: %BF = −12.937 + (0.696 x AC). Method 3 corresponded to an equation based on electrical bioimpedance that initially estimates FFM from body weight (BW), the 5-point BCS, and the body resistance index (R50 index), defined as the ratio between the square of the total body length (TL) and the resistance: R50 index = TL^2^ / R50. FFM was estimated using the equation: FFM = (0.624 x BW) − (1.579 x BCS) + (0.079 x R50 index) + 5.319. Subsequently, %BF was calculated as: %BF = ((BW − FFM) / BW) x 100. Method 4 corresponded to an alternative morphometric equation to estimate fat-free mass: FFM = (0.649 x BW) − (1.786 x BCS) + (0.114 x TL) + 0.188, and %BF was calculated in the same way: %BF = ((BW − FFM) / BW) x 100.

### Statistical analysis

Data were organized and analyzed using the free software Jamovi (version 2.3.28.0). Qualitative variables were described using relative frequencies, while quantitative variables are presented as mean ± standard deviation. A significance level of 5% was used. The data distribution was evaluated using the Shapiro–Wilk test. Subsequently, differences in electrical bioimpedance parameters and lipid profiles between obese and lean dogs were analyzed using an independent samples t-test. Given the small sample size and unequal distribution of the groups (4 obese vs. 13 lean), the t-test results were complemented with the calculation of the effect size (Cohen's d) in order to provide a measure of the magnitude of the observed differences independent of the sample size. The agreement between the body fat percentage (%BF) estimation methods was evaluated using Bland-Altman analysis to compare the different equations used, given that a reference method was not available (14). For each comparison, the bias and 95% limits of agreement were estimated, defined as the mean of the differences ±1.96 standard deviations. Complementarily, Lin's concordance correlation coefficient (CCC) was calculated for the same pairs of methods in order to quantify the degree of overall agreement between the %BF estimates. The agreement was interpreted according to criteria proposed in the literature (15), considering values <0.40 as poor agreement, between 0.40 and 0.75 as moderate, between 0.75 and 0.90 as substantial, and >0.90 as excellent.

Additionally, a post-hoc power analysis was performed for the comparisons of resistance, reactance, and phase angle between obese and lean dogs using G*Power software (version 3.1.9.7), employing an independent samples t-test for the difference in means with a 5% significance level.

#### Informed consent

Consent was obtained from all owners prior to the inclusion of the animals in the study.

#### Ethical considerations

Animal participation was carried out with the authorization of the professional ethics committee of the Universidad de Caldas; all procedures were non-invasive or minimally invasive and were conducted in compliance with animal welfare regulations and good veterinary clinical practices.

## Results

### Characteristics of the study population

Seventeen dogs were included, 35.3% females and 64.7% males. The mean age ± standard deviation was 6.56 ± 3.78 years. Regarding reproductive status, 94.1% of the animals were sterilized, while 5.9% were intact. As for the breed, 58.8% corresponded to mixed breed and the rest to breeds such as Yorkshire Terrier, Pitbull, Beagle, Cocker Spaniel, Australian Shepherd, and Pinscher.

According to the 5-point body condition score scale, 4 dogs (23.5%) were classified as obese (BCS 4.0–5.0/5) and 13 (76.5%) as lean (BCS 3.0–3.5/5). On the equivalent 9-point scale, the distribution was: BCS 5/9 (n = 5), BCS 6/9 (n = 8), BCS 7/9 (n = 1), BCS 8/9 (n = 2), and BCS 9/9 (n = 1). Regarding the breed distribution within each category, mixed-breed dogs predominated both in the lean group (9 of 13; BCS 5–6/9) and, to a lesser extent, in the obese group (1 of 4; BCS 8/9), while the other breeds (Yorkshire Terrier, Pitbull, Beagle, Cocker Spaniel, Australian Shepherd, and Pinscher) were sparsely distributed between both categories, without concentrating preferentially in either of them. The results are shown in Table 1.

**Table 1:** Distribution of body condition score (BCS) and breed in the study population.

**BCS (1/9)**	**BCS (1/5)**	**Breed**	**n**	**Body condition**
5	3.0	Mixed-breed	3	Lean
		Pinscher	2	

6	3.5	Mixed-breed	6	Lean
		Australian Shepherd	1	
		Pitbull	1	

7	4.0	Yorkshire Terrier	1	Obese

8	4.5	Cocker Spaniel	1	Obese
		Mixed-breed	1	

9	5.0	Beagle	1	Obese

Total			17	

**Note:** Dogs with a body condition score ≥ 7/9 were classified as overweight or obese. For the 5-point scale, dogs with a BCS ≥ 4/5 were considered overweight or obese.

### Characteristics of dietary management and physical activity

In relation to the type of feeding, 47.1% of the dogs consumed a commercial diet, 47.1% a mixed diet, and 5.9% a homemade diet. The daily amount of food provided presented high variability among individuals (268.0 ± 207.8 g/day).

Regarding feeding frequency, 70.6% were fed twice a day. In terms of physical activity level, 23.5% of the dogs presented high activity, 11.8% moderate activity, and 64.7% low activity.

### Morphometric measurements

The average values of the morphometric variables were: body weight 17.02 ± 10.94 kg, leg length 17.97 ± 5.03 cm, abdominal circumference 51.02 ± 16.22 cm, thoracic circumference 58.35 ± 15.49 cm, height at the withers 42.58 ± 10.04 cm, and total body length 59.1 ± 13.88 cm. The results are presented in Table 2.

**Table 2:** Morphometric results of the study population.

**Variable**	**Unit**	Mean ± SD*	**Min.**	**Max.**
Weight	kg	17.02 ± 10.94	3	39
Leg Length	cm	17.97 ± 5.03	10	27
Abdominal circumference	cm	51.02 ± 16.22	22.5	77
Thoracic circumference	cm	58.35 ± 15.49	34	87
Height	cm	42.58 ± 10.04	27	60
Total length	cm	59.11 ± 13.88	38	83

* SD: Standard deviation.

### Electrical bioimpedance parameters

The average values obtained by electrical bioimpedance were: resistance 252.35 ± 99.71 Ω, reactance 23.32 ± 10.02 Ω, and phase angle 5.72 ± 2.47°, as shown in Table 3.

**Table 3:** BIA parameters at 50 kHz in the total study population.

**Variable**	**Unit**	Mean ± SD*	**Minimum**	**Maximum**
Resistance	Ω	252.35 ± 99.71	115.50	435.80
Reactance	Ω	23.32 ± 10.02	9.00	40.70
Phase Angle	°	5.72 ± 2.47	1.80	10.00

* SD: Standard deviation.

When comparing obese and lean dogs, it was observed that obese animals presented, on average, a higher resistance value than lean ones (318.7 ± 113.3 Ω vs 231.9 ± 90.2 Ω) and a lower average phase angle value (4.6 ± 2.1° vs 6.1 ± 2.6°); however, these differences were not statistically significant (p > 0.05). The comparative results are presented in Table 4.

**Table 4:** Comparison of BIA variables between obese and lean dogs.

**Variable**	**Body Condition**	Mean ± SD*	t (df)**	**p-value**
Resistance (Ω)	Obese	318.70 ± 113.30	−1.59 (15)	0.13
	Lean	231.90 ± 90.20		
Reactance (Ω)	Obese	25.20 ± 12.50	−0.42 (15)	0.67
	Lean	22.70 ± 9.70		
Phase angle (°)	Obese	4.60 ± 2.08	1.04 (15)	0.31
	Lean	6.07 ± 5.56		

* SD: Standard deviation df: degrees of freedom in independent samples t-test.

### Lipid profile

Total cholesterol presented a mean of 232.94 ± 42.17 mg/dL, while HDL cholesterol showed a mean of 168.06 ± 33.22 mg/dL; both variables presented a normal distribution (p > 0.05).

No statistically significant differences were observed in total cholesterol between obese and lean dogs (p = 0.119). In contrast, HDL cholesterol was significantly higher in lean dogs than in obese dogs (177.46 ± 31.62 mg/dL vs 137.50 ± 16.01 mg/dL; p = 0.030). These results are presented in Table 5.

**Table 5:** Cholesterol values in obese and lean dogs.

**Variable**	**Body Condition**	**Mean ± SD**	**t (df)**	**p-value**
Total	Obese	204.00 ± 37.71	1.65 (15)	0.11
Cholesterol	Lean	241.85 ± 41.28		

HDL	Obese	137.50 ± 16.01	2.39 (15)	0.03*
Cholesterol	Lean	177.46 ± 31.62		

* SD: Standard deviation df: Degrees of freedom in independent samples t-test.

* Statistical significance (p < 0.05).

### Agreement between body fat percentage estimation methods

**Figure 2.**
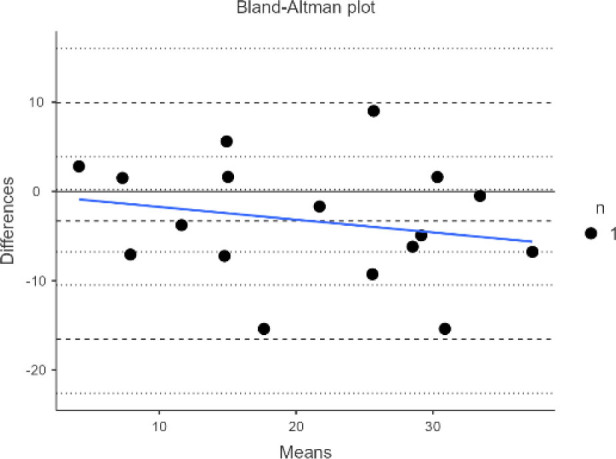
Agreement between Method 1 vs Method 2.

**Figure 3.**
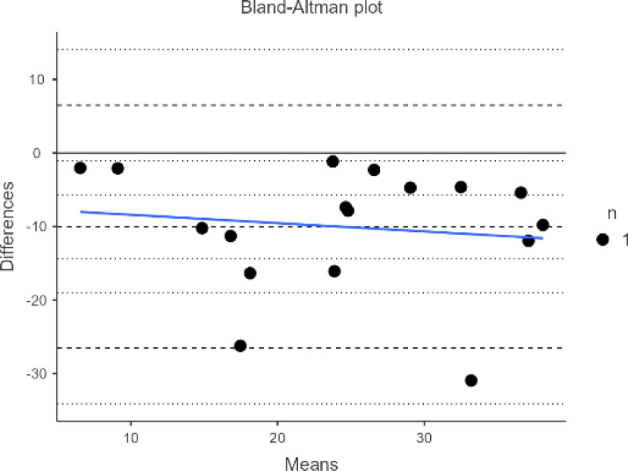
Agreement between Method 1 vs Method 3.

**Figure 4.**
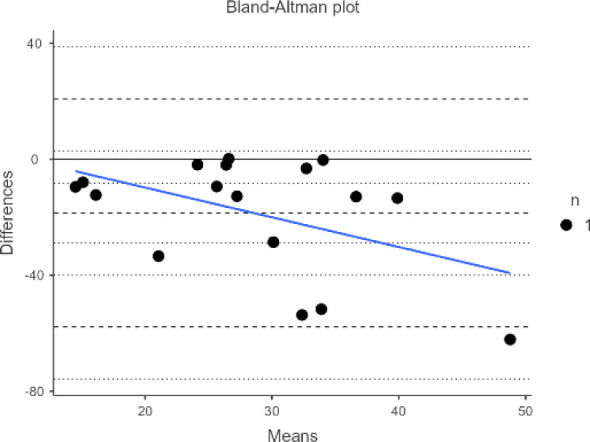
Agreement between Method 1 vs Method 4.

**Figure 5.**
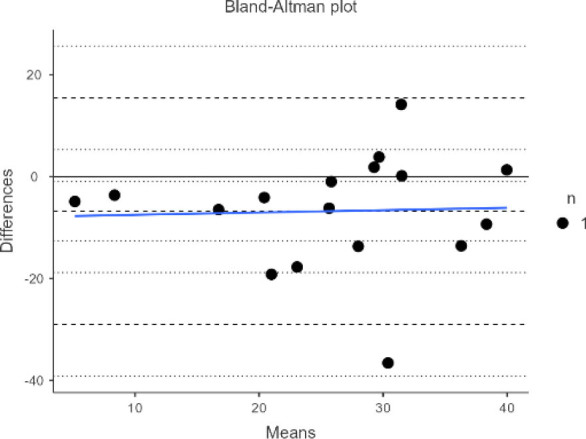
Agreement between Method 2 vs Method 3.

**Figure 6.**
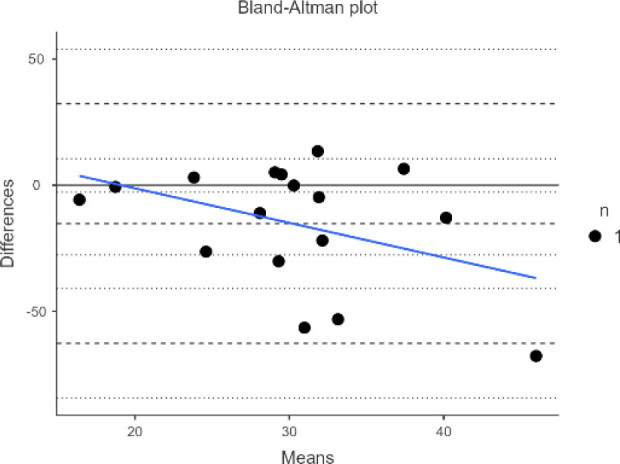
Agreement between Method 2 vs Method 4.

**Figure 7.**
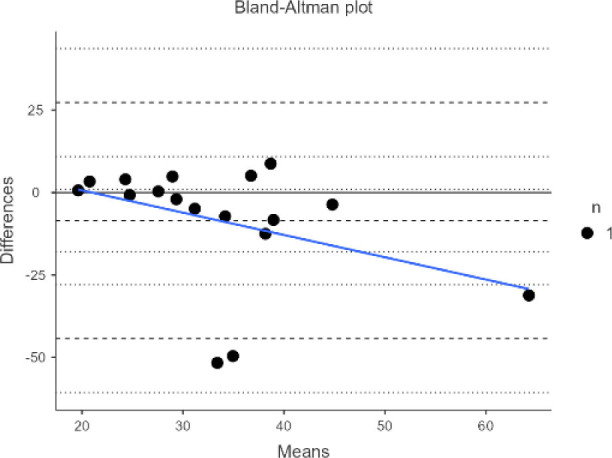
Agreement between Method 3 vs Method 4.

The Bland-Altman analysis evidenced differences between the %BF estimation methods. Biases ranged from −3.3 to −18.5, with wide limits of agreement in all comparisons, indicating low consistency between methods (Table 6). The greatest discrepancies were observed in the comparisons involving method 4, particularly against method 2, where the widest range of dispersion was evidenced (Figures 2 to 7).

**Table 6:** Bland–Altman analysis between %bf estimation methods.

**Method comparison**	**Bias (%)**	**Lower limit (95%)**	**Upper limit (95%)**
Method 1 vs Method 2	−3.30	−16.50	9.90
Method 1 vs Method 3	−10.00	−26.60	6.50
Method 1 vs Method 4	−18.50	−57.80	20.80
Method 2 vs Method 3	−6.70	−29.00	15.50
Method 2 vs Method 4	−15.20	−62.70	32.20
Method 3 vs Method 4	−8.50	−44.30	27.30

**Note:** Bias corresponds to the mean of the differences between methods. Limits of agreement are calculated as bias ± 1.96 standard deviations of the differences. No reference method was used.

### Correlation-agreement between methods

Lin's concordance correlation coefficient (CCC) showed a moderate agreement between method 1 and method 2 (CCC = 0.75; 95% CI: 0.47–0.89), while the remaining comparisons evidenced low or negative agreement, with wide confidence intervals reflecting the uncertainty associated with the small sample size.

Particularly, the comparisons between methods based on bioimpedance and morphometry showed low agreement, highlighting values close to zero or negative, suggesting poor equivalence between methods. The results are presented in Table 7.

**Table 7:** Lin's concordance correlation coefficient (ccc) between %bf estimation methods.

**Method comparison**	**CCC**	**Lower 95% CI**	**Upper 95% CI**
Method 1 vs Method 2	0.75	0.47	0.89
Method 1 vs Method 3	0.45	0.14	0.68
Method 1 vs Method 4	−0.04	−0.26	0.17
Method 2 vs Method 3	0.40	−0.005	0.69
Method 2 vs Method 4	−0.29	−0.56	0.03
Method 3 vs Method 4	0.11	−0.26	0.46

**Note.** Lin's concordance correlation coefficient: Values close to 1 indicate high agreement, while values close to 0 or negative indicate low or no agreement.

## Discussion

Canine obesity represents a growing challenge in small animal medicine, not only due to its high prevalence but also because of the difficulty in accurately quantifying adiposity under clinical conditions. Although body weight and visual assessment remain widely used tools, their limited ability to discriminate changes in body composition highlights the need for more objective methods [16].

In this context, the present study focused on evaluating the agreement between different indirect methods for estimating body fat percentage. The most relevant finding was the low agreement between the evaluated methods, evidenced both by the wide limits of agreement in the Bland-Altman analysis and by the low Lin's concordance correlation coefficients in most comparisons [14,15]. This result indicates that the methods are not interchangeable and that they can generate substantially different estimates in the same individual.

The highest agreement observed between the morpho-metric methods (CCC = 0.759) was expected, given that they share a similar structural basis [5]. In contrast, methods 3 and 4, derived from Rae et al. [3], showed inconsistent behavior in this population, including low or negative agreements, particularly in the case of the exclusively morpho-metric method (Method 4). These findings suggest that equations developed under controlled conditions and in homogeneous populations may lose validity when applied in real clinical scenarios with dogs presenting high morphological variability.

In particular, methods 3 and 4 were originally derived in populations of purebred dogs, under sedation, and using DEXA as a reference method. Their application in conscious, mixed-breed, client-owned animals, without a prior cross-validation process, may introduce a systematic error that cannot be quantified with the data from the present study, and which could explain, at least in part, the low agreement observed in the comparisons involving these methods.

The observed differences can be explained by multiple factors. First, the original equations were developed under highly standardized conditions, including anesthesia or sedation and prolonged fasting protocols, which differ from the conditions of the present study, where measurements were performed in conscious animals, with changing temperature and humidity conditions, and with different hydration levels, making the physiological control of the patients difficult. Second, the morphological heterogeneity of the evaluated population, characterized by a high percentage of mixed-breed dogs, introduces an additional source of variability that is not considered in the available predictive equations [3,8].

Furthermore, the use of a fixed distance of 7 cm between the electrodes introduces a considerable geometric confounding variable. In small dogs, this distance represents a significantly larger portion of the anatomical segment compared to large dogs, which alters the cross-sectional pathway of the tissue and highlights the need to standardize the spacing proportionally to the animal's size in future studies.

Despite the limited agreement in the estimation of %BF, the raw electrical parameters obtained by bioimpedance showed physiologically coherent behavior with body condition. Obese dogs presented higher resistance and a lower phase angle, reflecting a lower proportion of total body water and possible alterations in cellular integrity or fluid distribution [11,17], although these differences did not reach statistical significance (p > 0.05). In humans, it is common to observe lower phase angle values in overweight and obese individuals compared to healthy controls of the same age and sex, particularly in those with metabolically unfavorable obesity [18].

However, this evidence comes from human medicine, and its extrapolation to canines should be done with caution, given the differences in body composition, adipose tissue distribution, and measurement protocols between the two species.

Regarding the lipid profile, the results did not evidence a clear pattern of dyslipidemia associated with obesity. The absence of significant differences in total cholesterol and the reduction of HDL cholesterol in obese dogs should be interpreted with caution, especially considering the small size of the obese animal group. Nevertheless, these findings are consistent with previous reports suggesting that canine obesity is not always associated with detectable lipid alterations under basal conditions, reinforcing the metabolic complexity of this condition [19].

The variables collected through the survey showed a pattern consistent with that described in the literature, highlighting a sedentary lifestyle as a predominant factor in obese dogs [20]. However, given the descriptive design of the study, these results should be considered only as an epidemiological context and not as evidence of causal relationships.

This study presents several limitations that must be considered. The absence of a reference method precludes evaluating the accuracy of the compared methods, restricting the interpretation and the agreement analysis. Furthermore, the inherent variability in measurement conditions—such as the hydration level, which was not standardized or controlled by a specific protocol prior to measurement, ambient temperature, and humidity—may have contributed to the observed results. Likewise, the fixed distance of 7 cm between the current and voltage electrodes, although consistent with previously described protocols [8], was not adjusted proportionally to the animals' body size, which could have introduced a geometric confounding variable in a population with weights ranging from 3 to 39 kg. In smaller dogs, this distance could have exceeded the anatomical limits of the evaluated segment or significantly modified the pathway of the current through the tissues, whereas in larger dogs it represented a minimal proportion of the limb's length. This limitation must be considered when interpreting the absolute values obtained for resistance, reactance, and phase angle, and it is recommended that future studies employ an electrode distance proportional to body size or referenced to specific anatomical landmarks, rather than a fixed linear measurement.

Additionally, the small sample size increases the probability of a Type II error. Therefore, the lack of statistical significance in the comparisons of resistance, reactance, and phase angle should not be interpreted as evidence of an absence of biological difference, but rather as an inherent limitation of the available sample size. Consequently, the present study should be understood as an exploratory and hypothesis-generating work, whose findings require confirmation in larger cohorts before suggesting clinical applicability.

The post-hoc power analysis revealed that the effect sizes (Cohen's d) calculated from the observed means and standard deviations corresponded to a large magnitude for resistance (d = 0.85), moderate for phase angle (d = 0.63), and small for reactance (d = 0.22). However, the statistical power achieved was only 0.28, 0.18, and 0.07, respectively, values well below the conventional threshold of 0.80 recommended to consider a study adequately powered. These results are particularly relevant for resistance and phase angle, where a moderate to large effect size, coupled with low statistical power, suggests that the observed differences between groups could have biological relevance and that the absence of statistical significance (Table 3) is likely due to a Type II error derived from the small size of the obese dog group (n = 4), rather than a true physiological equivalence between both groups. In contrast, for reactance, both the small effect size and very low power suggest that the difference between groups, if it exists, would be of limited magnitude. Taken together, these results reinforce the need to interpret the findings of this study as exploratory and to prioritize larger and more balanced sample sizes, particularly for the obese animal group, in future research.

Taken together, the findings of this study support two main conclusions: first, that the evaluated morphometric and bioelectrical equations are not interchangeable with each other in this population; and second, that the raw bioelectrical parameters show a preliminary, although not statistically significant, pattern of association with body condition. These results should be interpreted as hypothesis-generating rather than sufficient evidence for immediate clinical implementation.

Despite these limitations, the results provide a relevant point for clinical practice: the lack of agreement among the indirect methods for estimating body fat, BCS, and morphometry, implies that their isolated use can lead to erroneous interpretations of the dog's nutritional status. In this regard, electrical bioimpedance, morphometric equations, and BCS should be considered as complementary tools to each other and not as substitutes.

Finally, these findings highlight the need to validate body composition assessment methods in specific populations, especially in contexts with high morphological diversity such as the Colombian canine population. The development of adapted predictive equations and the standardization of measurement protocols represent fundamental steps to improve diagnostic accuracy in veterinary clinical nutrition.

## Conclusion

The evaluated indirect methods for estimating body fat percentage in dogs showed low agreement, indicating that they are not interchangeable in clinical practice. The morphometric equations and those derived from bioimpedance produced inconsistent estimates, particularly when applied in real clinical conditions, in patients of different breeds and body conditions. Despite this, the raw electrical parameters of bioimpedance, such as resistance, reactance, and phase angle, showed physiological behavior consistent with body condition. This suggests a potential utility of their direct interpretation, rather than the use of predictive equations not validated for the evaluated population [17]; however, lacking a reference method, these findings must be considered preliminary and do not constitute evidence of diagnostic accuracy. Thus, future studies using reference methods such as DEXA to confirm these findings are justified.

Taken together, these findings indicate that the estimation of adiposity in dogs using indirect methods should be approached with caution, prioritizing an integrated approach that combines clinical assessment, morphometry, and bioelectrical parameters.

## References

[1] Tvarijonaviciute A, Muñoz-Prieto A, Martinez-Subiela S, Pastorinho MR, Sousa ACA (2020). Obesity in Humans and Dogs: Similarities, Links, and Differences. Pets as Sentinels, Forecasters and Promoters of Human Health.

[2] Preet GS, Turkar S, Gupta S, Kumar S (2021). Dog obesity: Epidemiology, risk factors, diagnosis and management: A review paper. Pharma Innovation.

[3] Rae LS, Vankan DM, Rand JS, Flickinger EA, Ward LC (2016). Measuring body composition in dogs using multifrequency bioelectrical impedance analysis and dual energy X-ray absorptiometry. The Veterinary Journal.

[4] Yaguiyan-Colliard L, Daumas C, Bousbiat S, Jaffrin M, Cardot P, Grandjean D (2015). Indirect prediction of total body water content in healthy adult Beagles by single-frequency bioelectrical impedance analysis. American Journal of Veterinary Research.

[5] Mawby DI, Bartges JW, d'Avignon A, Laflamme DP, Moyers TD, Cottrell T (2004). Comparison of Various Methods for Estimating Body Fat in Dogs. Journal of the American Animal Hospital Association.

[6] Fosbøl MØ, Zerahn B (2015). Contemporary methods of body composition measurement. Clin Physio Funct Imaging.

[7] Ward LC, Brantlov S (2023). Bioimpedance basics and phase angle fundamentals. Rev Endocr Metab Disord.

[8] Yaguiyan-Colliard L, Daumas C, Nguyen P, Grandjean D, Cardot P, Priymenko N (2015). Evaluation of total body water in canine breeds by single-frequency bioelectrical impedance analysis method: specific equations are needed for accuracy. BMC Res Notes.

[9] Agudelo Giraldo L, Narváez Solarte W (2019). Prevalencia de la obesidad en Canis lupus familiaris Linnaeus, 1758 (Carnivora: Canidae) en Manizales, Colombia. Bol cient mus hist nat univ caldas.

[10] Cita NV, Acero RA, Gallego LS, Villalba D (2024). Presence of overweight and obesity in canines (Canis lupus familiaris) and its risk factors in the North of Bogotá. Rev Med Vet Zoot.

[11] Li Z, Mandour AS, Farag A, Xu T, Terai K, Shimada K (2025). Phase Angle as a Non-Invasive Biomarker of Fluid Overload in Canine Right Heart Failure: A Bioelectrical Impedance Approach. Animals.

[12] Rae LS, Rand JS, Ward LC (2024). Measuring body composition in dogs using bioelectrical impedance spectroscopy. The Veterinary Journal.

[13] Laflamme DP (2006). Understanding and Managing Obesity in Dogs and Cats. Veterinary Clinics of North America: Small Animal Practice.

[14] Giavarina D (2015). Understanding Bland Altman analysis. Biochem Med.

[15] Camacho-Sandoval J (2008). Coeficiente de concordancia para variables continuas [Internet].

[16] Stone R, Berghoff N, Steiner JM, Zoran D (2009). Use of a Bioelectric Impedance Device in Obese and Lean Healthy Dogs to Estimate Body Fat Percentage. Veterinary Therapeutics.

[17] Nisini N, Corda A, Birettoni F, Miglio A, Antognoni MT (2024). Bioelectrical Impedance Analysis (BIA) detects body resistance increase in dogs undergoing blood donation. Vet Res Commun.

[18] Cancello R, Brunani A, Brenna E, Soranna D, Bertoli S, Zambon A (2023). Correction to: Phase angle (PhA) in overweight and obesity: evidence of applicability from diagnosis to weight changes in obesity treatment. Rev Endocr Metab Disord.

[19] Jeusette IC, Lhoest ET, Istasse LP, Diez MO (2005). Influence of obesity on plasma lipid and lipoprotein concentrations in dogs.

[20] Cesare San Martín ELE, Echeverría-Jaque CL, Macuer-Guzmán JE (2024). Análisis de la relación entre la obesidad de tutores, sus perros y One Welfare. Una Revisión bibliográfica. epsir.

